# A Newly Identified LncRNA LncIMF4 Controls Adipogenesis of Porcine Intramuscular Preadipocyte through Attenuating Autophagy to Inhibit Lipolysis

**DOI:** 10.3390/ani10060926

**Published:** 2020-05-26

**Authors:** Yunmei Sun, Rui Cai, Yingqian Wang, Rui Zhao, Jin Qin, Weijun Pang

**Affiliations:** Laboratory of Animal Fat Deposition and Muscle Development, Key Laboratory of Animal Genetics, Breeding and Reproduction of Shaanxi Province, College of Animal Science and Technology, Northwest A&F University, Yangling, Shaanxi 712100, China; sunyunmei@nwafu.edu.cn (Y.S.); cairui1663@nwafu.edu.cn (R.C.); Yingqianwang@126.com (Y.W.); ZR970510@163.com (R.Z.); qinjin19921026@sina.com (J.Q.)

**Keywords:** pork quality, lncIMF4, intramuscular preadipocyte, differentiation, autophagy

## Abstract

**Simple Summary:**

Compared with lean-type pigs, the intramuscular fat content of fat-type Bamei pigs was greater. LncRNA, as a vital regular, plays an important role in numerous biological processes. However, there were a few studies on the role of lncRNAs during IMF development in pigs. Based on these, lncRNA sequencing in intramuscular adipocytes was performed to explore the effects of lncRNA on intramuscular fat deposition. RNA sequencing analysis of intramuscular adipocyte from Bamei pig (fat-type) and Yorkshire pig (lean-type) indicated that, a novel lncRNA, lncIMF4, was associated with intramuscular adipogenesis. In addition, further researches showed that knockdown lncIMF4 promoted proliferation and adipogenic differentiation of porcine intramuscular adipocytes, whereas inhibited autophagy. Moreover, knockdown lncIMF4 facilitated intramuscular adipogenesis through attenuating autophagy to repress the lipolysis. Our findings will contribute to better understand the mechanism of lncRNA controlling adipogenesis in pig. Furthermore, it also provides a new perspective to study the role of lncRNA in regulating porcine intramuscular adipogenesis for promoting pork quality.

**Abstract:**

Intramuscular fat (IMF) is implicated in juiciness, tenderness, and flavor of pork. Meat quality of Chinese fat-type pig is much better than that of lean-type pig because of its higher IMF content. LncRNA is a vital regulator that contributes to adipogenesis. However, it is unknown about the regulation of lncRNA on IMF content. Here, by RNA sequence analysis of intramuscular adipocyte from Bamei pig (fat-type) and Yorkshire pig (lean-type), we found that a novel lncRNA, lncIMF4, was associated with adipogenesis. LncIMF4, abundant in adipose, differently expressed along with intramuscular preadipocyte proliferation and differentiation. Meanwhile, it is located both in cytoplasm and nucleus. Besides, lncIMF4 knockdown promoted proliferation and differentiation of porcine intramuscular preadipocytes, whereas inhibited autophagy. Moreover, lncIMF4 knockdown facilitated intramuscular adipogenesis through attenuating autophagy to repress the lipolysis. Our findings will contribute to understand better the mechanism of lncRNA controlling intramuscular adipogenesis for promoting pork quality.

## 1. Introduction

Adipose tissue is essential for animals. It plays a vital role in the composition of living organisms and various metabolic processes. Among them, intramuscular adipose relates to meat quality. Moderate intramuscular fat content can increase the tenderness and flavor of pork. The content of intramuscular fat (IMF) and its fatty acid composition play an important role in meat quality, affecting the sensory properties (juiciness, flavor, and tenderness), and nutritional value of meat [1]. The positive effects of IMF related to the quality of meat have been confirmed in pork [2], mutton [3], and beef [4]. The content depends not only on the amount of precursor fat cells converted to mature IMF cells but also on the deposition of lipid droplets in the IMF cells and lipid droplets in the myocytes [5].

LncRNA, a long noncoding RNA, participates in multiple life processes. They participated in many cellular biological processes, such as proliferation, differentiation, and apoptosis, by regulating the expression of their target genes [6,7,8]. There is some evidence demonstrated that lncRNAs regulated adipogenesis through multiple mechanisms. Hundreds of lncRNAs were involved in the regulatory network of adipogenesis [9,10]. More and more researchers are paying attention to study the influence of lncRNA on pig fat deposition and exploring its regulation for increasing meat quality. At present, many studies about lncRNA sequencing in adipose tissue from different pig breeds were performed. These sequencing analyses illustrated that many lncRNAs were involved in the development of porcine adipose tissue [11,12,13]. However, there were a few studies on the role of lncRNAs during IMF development in pigs. This exciting area needs to be further researched.

Autophagy is a process of phagocytizing its cytoplasmic protein or organelle and coating it into vesicles and fusing with lysosomes to form autophagosomes. Autophagy degrade the contents by autophagosomes to regulate cell metabolism. In addition, it updates to specific organelles; cell autophagy, like apoptosis and cell senescence, is an important biological phenomenon involved in the development and growth of organisms. Macroautophagy, is the best understood among the known autophagic pathways. It is characterized by forming a double-membrane structure called the autophagosome that fuses with the lysosome [14]. The study found that there was also the occurrence of macroautophagy in adipocytes. The lysosome contains various hydrolases; therefore, autophagy can degrade multiple cytoplasmic components and provide the resultant molecular building blocks, such as amino acids, glucose, nucleotides, and fatty acids [15]. Experiments in mouse models have shown that autophagy is required to maintain the levels of amino acids and glucose in blood and tissues of neonatal and adult mice during fasting [16,17,18]. Therefore, in order to investigate the mechanism of lncRNA on regulating adipose development, studies about the relationship between lipogenesis and autophagy require further exploration.

In our previous studies, we performed lncRNA sequencing in preadipocytes of porcine longissimus dorsi muscle during differentiation [19]. The data showed that lncIMF4 is a novel lncRNA. It was differentially expressed in different time groups. This inspired that lncIMF4 may be a potential target for adipogenesis. Additionally, bioinformatics analysis predicted that lncIMF4 participated in many biological processes including cell proliferation, differentiation, and autophagy. Therefore, this research on the function of lncIMF4 in intramuscular adipocytes was performed to better understand the mechanism of lncRNA controlling intramuscular adipogenesis for promoting pork quality.

## 2. Materials and Methods

### 2.1. Animal

Animal samples used in this study were approved by the Animal Care and Use Committee of Northwest A&F University. To investigate the expression pattern of lncIMF4 in pig, tissue samples from heart, liver, spleen, lungs, kidney, fat, and skeletal muscle (longissimus dorsi) of Bamei pigs at 3 days old (n = 4) and 180 days old (n = 4) were collected from experimental farm of Northwest A&F University (Yangling, China). All tissues for expression profile were immediately frozen in liquid nitrogen and then were kept at −80 °C until RNA isolation.

### 2.2. Cell Culture

The experiments refer to in vitro intramuscular adipocytes from Bamei and Yorkshire pigs. Intramuscular preadipocytes were isolated from longissimus dorsi muscle (LD) of 3-day-old piglets as described previously [19]. In brief, we used 0.2% collagenase I (270 U/mg; Gibco, Carlsbad, CA, USA) to digest samples at 37 °C in the water bath shaker for 2 h. Then, samples were sequentially filtered through 70 and 200 mesh filters to separate the cells. After washing twice with DMEM/F12, cells were seeded in dishes containing DMEM/F12 medium with 10% fetal bovine serum (Gibco, Australia). After 1.5 h, we rinsed off unattached cells. When density of cells reaches to approximately 100%, we used the cocktail method to induce adipocytes differentiation. The four differentiation stages were observed when cells were cultured in differentiation medium for 0 d (undifferentiated), 2 d (early differentiated), 4 d (middle differentiated), and 8 d (last differentiated). The autophagy was induced by a serum-free medium for 3 h after 6-day differentiation. Until that, preadipocytes were cultured in DMEM/F12 to proliferate. Briefly, we used OPTI to dilute negative control (NC) (or lncIMF4 siRNA to a concentration of 50 nM/mL), kept still for 5 min, then added Roche transfection reagent, gently blew, and further kept for 20 min. This mixed OPTI was added to the new medium.

### 2.3. Cell Transfection

For research on proliferation, 50 nM siRNA or negative control (NC) were transfected into cells by X-tremeGENE siRNA Transfection Reagent (Roche, San Francisco, USA) and Opti-MEM (Gibco, Grand Island, USA) when the density was 40%. After transfection for 24 h, the cells were harvested. For differentiation, when the density reached 80%, siRNA or NC were transfected, and differentiation medium was replaced with growth medium when the cells reached fusion. The siRNA and NC were obtained from RiboBio (Guangzhou, China).

### 2.4. Hematoxylin–Eosin Staining

The longissimus dorsi muscle tissue was taken from the fifth to sixth lumbar vertebrae of the 180-day-old Bamei pigs (n = 6) and the Yorkshire pigs (n = 6) and placed in the fixative. Longissimus dorsi muscle (LM) tissues were dehydrated by alcohol. The dehydrated tissues were made into paraffin sections. For HE staining, the sections were deparaffinized with xylene for 10 min. Then, the sections were washed with the distilled water and then were first placed in an aqueous solution of hematoxylin for several minutes. Then the sections were immersed in acid water and ammonia water for color separation for several seconds, respectively. Next, the sections were rinsed under the running water for 1 h, and then were immersed into distilled water for a while. Slices were dehydrated in 70% and 90% alcohol for 10 min, respectively, and then were dyed in eosin staining solution for 2–3 min. The stained sections were dehydrated with pure ethanol, and the sections were made transparent by xylene. The transparent slices were mounted with Canada Balsam and covered with a coverslip. Sample images were captured using a Nikon TE2000 microscope (Nikon, Tokyo, Japan).

### 2.5. Fluorescence in Situ Hybridization

Cell slide was placed on the bottom of the 12-well plate; cells were washed for 5 min and then were fixed at room temperature for 10 min with 4% paraformaldehyde. Briefly, 200 uL prehybrid solution was added to each well and they were blocked at 37 °C for 30 min. For prehybridization, the hybridization solution was preheated at 37 °C. To protect it from the light, 2.5 μL 20 uM lncRNA FISH Probe Mix stock solution or internal reference FISH Probe Mix stock solution was added to 100 μL hybridization solution. The prehybridization solution in each well was discarded and 100 μL of the probe hybridization solution containing the probe was added; the light was avoided, and then it was hybridized overnight at 37 °C. DNA staining is protected from light. Further, staining was done with DAPI staining solution for 10 min, avoiding light, and the cells were washed 3 times for 5 min each time. The cell slide was carefully removed from the well in dark and fixed on the loaded slide with a sealing tablet for fluorescence detection.

### 2.6. Cytoplasmic and Nuclear RNA Extraction

This method is referred to a previous research [20]. For the extraction of cytoplasmic and nuclear RNA fraction, intramuscular adipocytes were collected after 6-day differentiation. Cells were washed with PBS, suspended in lysis buffer (10 mM NaCl, 2 mM MgCl_2_, 10 mM pH 7.8 Tris-HCL, 5 mM DTT, and 0.5% Igepal CA 630), and then incubated on ice for 5 min. After centrifuging at 8000 rpm for 5 min, the supernatant was transferred to a new microcentrifuge tube subjected to cytoplasmic RNA extraction, while the pellet was resuspended with lysis buffer and subjected to nuclear RNA extraction. For the RNA extraction, the fractions were first incubated with Proteinase K (10 mg/mL) at 37 °C for 20 min and then mixed with TRIzol. RNA was separated by chloroform and precipitated by ethanol with 3 M sodium acetate (pH 5.2, 1/10 volume). The extracted RNA was dissolved into ddH_2_O and used for reversed transcribed and real-time PCR analysis.

### 2.7. RNA Extraction and Real-Time PCR

Total RNA was isolated by TRIzol reagent (Takara, Otsu, Japan). Afterwards, the reverse transcription of RNA was performed using kits (Takara, Otsu, Japan), following the manufacturer’s instructions. Bio-Rad iQTM5 (Bio-Rad, Hercules, CA, USA) was used to perform RT-qPCR. Expression level of the listed genes and lncIMF4 were related to that of β-tubulin. The information of primers is shown in Table 1.

### 2.8. Western Blotting

The protocol of western blot was followed according to a previous study [10]. Briefly, adipocytes were split by radioimmunoprecipitation assay (RIPA) buffer (Beyotime, China) by adding protease inhibitor (Pierce, WA, USA). The total protein sample was separated in the SDS-polyacrylamide gel. Then, it was transferred into a PVDF membrane (Millipore, Bedford, MA, USA). Next, the membrane was blocked in 5% defatted milk for 2 h. After that, the membrane was incubated with primary antibodies at 4 °C overnight followed by a secondary antibody at room temperature for 1.5 h. The antibodies Cyclin B, Cyclin D, Cyclin E, PPARγ, and AP2 were purchased from Santa Cruz (CA, USA); C/EBPα and SREBP-1 were purchased from Abcam; p62, LC, ATGL, and HSL were purchased from CST (Boston, MA, USA); and β-tubulin was purchased from Sungene Biotech (Shanghai, China).

### 2.9. Cell Counting Kit (CCK8) and EdU Assays

The protocol of cell counting (CCK8) and EdU assays were performed following a previous study [6]. Adipocytes were cultured in 96-well plates with 2.5 × 103 cells per well. Cells were treated with NC or siRNA. The proliferation rates were tested by the Cell Counting Kit 8 (CCK-8) following manufacturer’s instructions. For EdU assay, cells were incubated with EdU for 2 h after transfecting them with NC or siRNA. Subsequently, sample images were captured using a Nikon TE2000 microscope (Nikon, Tokyo, Japan).

### 2.10. Flow Cytometry

Cells were seeded in 6-well plates with 4 × 105 cells per well. After 24 h, cells were transfected with NC or siRNA. Next, cells were washed three times with PBS and fixed with 70% alcohol overnight at −20 °C. Cells were then treated with 1 mg/mL RNase at 37 °C for 40 min before being stained with 50 mg/mL propidium iodide (PI) at 4 °C for 1 h. Lastly, samples were detected with FACSCalibur flow cytometry (Franklin Lakes, NJ, USA).

### 2.11. Oil Red O Staining

Oil Red O Staining was performed according to a previously published method [12]. After being fixed in 4% paraformaldehyde solution, cells were incubated with 0.5% Oil Red O for 30 min and washed three times with PBS; adipocytes were visualized by phase-contrast microscopy (Tokyo, Japan). Oil Red O dissolved in lipid droplets was extracted with 100% isopropanol and its relative concentrations were determined by measuring the absorbance at 510 nm.

### 2.12. Monodansylcadaverine (MDC) Staining

The cells were digested with trypsin and centrifuged, and the cell pellet was collected. Then the cells were washed once with wash buffer and the supernatant was discarded. Next, the cells were resuspended by adding the appropriate amount of wash buffer to adjust the cell density to 10. Precisely, 90 uL of cell suspension was taken into the EP tube and 10 uL of MDC solution was added and mixed gently. The solution was stained at room temperature for 30 min, and the cells were collected by centrifugation. Then, the cells were washed twice with wash buffer, 300 uL DAPI solution was added to it, and stood still at room temperature. After 5 min, the cells were washed 3 times with PBS. The cells were resuspended in the collection buffer, the resuspended droplets were added to the slide, and the coverslip was attached. The slide was observed under a fluorescence microscope, and cells were counted and photographed. Autophagy staining detection kit was purchased from Solarbio.

### 2.13. Statistical Analysis

The diagrams were created by GraphPad Prism 6.0. SEM represents the variation between sample means. Group differences were analyzed with Student’s *t* test or one-way ANOVA using PASW Statistics 20 (SPSS, Chicago, IL, USA) (*, *p* < 0.05; **, *p* <0.01).

## 3. Results

### 3.1. LncIMF4 May be A Novel LncRNA Implicated in Intramuscular Fat Deposition

**Figure 1:**
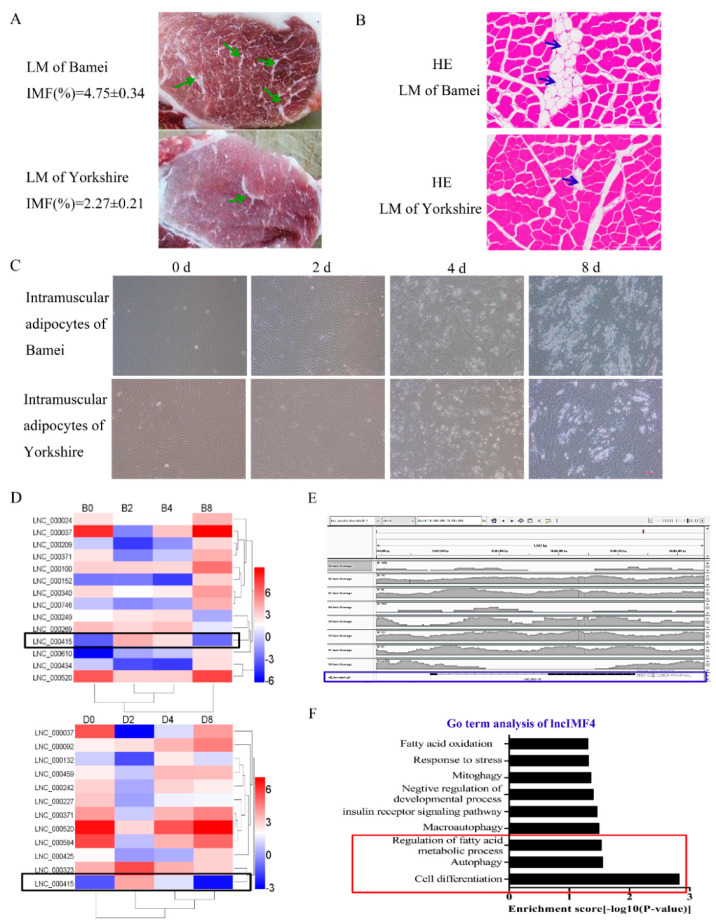
LncIMF4 may be a novel lncRNA implicated in intramuscular adipogenesis. (**A**) The intramuscular fat (IMF) content of longissimus dorsi muscle (LM) between Bamei and Yorkshire were analyzed by Soxhlet extraction method. (**B**) Hematoxylin–eosin (HE) staining of LM between Bamei and Yorkshire. (**C**) Porcine intramuscular adipocytes at 0, 2, 4, and 8 d after inducing differentiation (cell density reached 100%). (**D**) Heatmap depicting long noncoding RNA (lncRNA) having at least 2-fold change in intramuscular adipocytes between fat-type and lean-type pig at four differentiation stages; black fragments denote lncIMF4. (**E**) The alignment track of lncIMF4. (**F**) GO term of lncIMF4. The results were representative of means ± SEM of three independent experiments.

The meat quality of Bamei pig is better than Yorkshire pig probably highly influenced by the higher content of intramuscular adipocytes in Bamei pig. (Figure 1A,B). The lipid content in intramuscular adipocytes of Bamei pig was also more than Yorkshire (Figure 1C). Comparison of lncRNA sequences was performed between intramuscular adipocytes of Bamei pig and Yorkshire pig. The results showed that the level of lncIMF4 was higher in large white pig, and it presented rising first then falling pattern (Figure 1D). In addition, the alignment track showed that lncIMF4 is an unannotated lncRNA in the pig genome (Figure 1E). Interestingly, the result of Gene Ontology (GO) term analysis demonstrated that it was related with lipolysis and autophagy (Figure 1F). Therefore, further research was carried out following these pathways.

### 3.2. The Expression Pattern of LncIMF4 in Pig and Its Subcellular Location in Adipocyte

**Figure 2:**
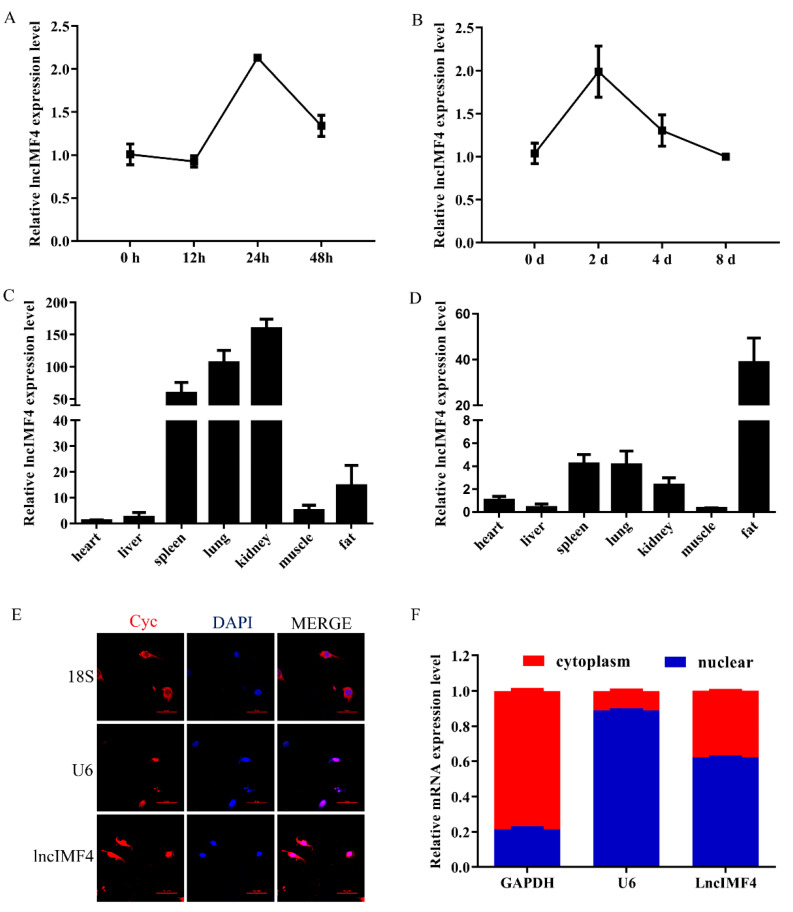
The expression pattern of lncIMF4 in pig and its subcellular location in adipocyte. (**A**) The levels of lncIMF4 during porcine intramuscular adipocytes proliferation. (**B**) The levels of lncIMF4 during adipocytes differentiation. (**C**,**D**) The levels of lncIMF4 in heart, liver, spleen, lung, kidney, muscle, and fat from 3-day-old piglets (**C**) and 180-day-old pig (**D**). (**E**) Subcellular localization of lncIMF4. (**F**) LncIMF4 expression in cytoplasm and nucleus. The results were representative of means ± SEM of three independent experiments.

Based on the above results, we supposed that lncIMF4 was related to IMF content. We detected the levels of lncIMF4 during intramuscular adipocytes proliferation and differentiation. The expression patterns both showed a trend of rising first and then decreasing, reaching a peak on the second day, which was consistent with the sequencing results (Figure 2A,B). Tissue expression results demonstrated that lncIMF4 is highly expressed in adipose tissue, not only in 3-day-old piglets (Figure 2C) but also in 180-day-old pig (Figure 2D). To confirm the localization of lncIMF4 in intramuscular adipocytes, we performed the fluorescence in situ hybridization, showing that lncIMF4 was localized in both nucleus and cytoplasm (Figure 2E). The relative expression levels of LncIMF4 in the nucleus and cytoplasm were consistent with it (Figure 2F).

### 3.3. LncIMF4 Knockdown Promoted the Proliferation of Porcine Intramuscular Preadipocytes

**Figure 3:**
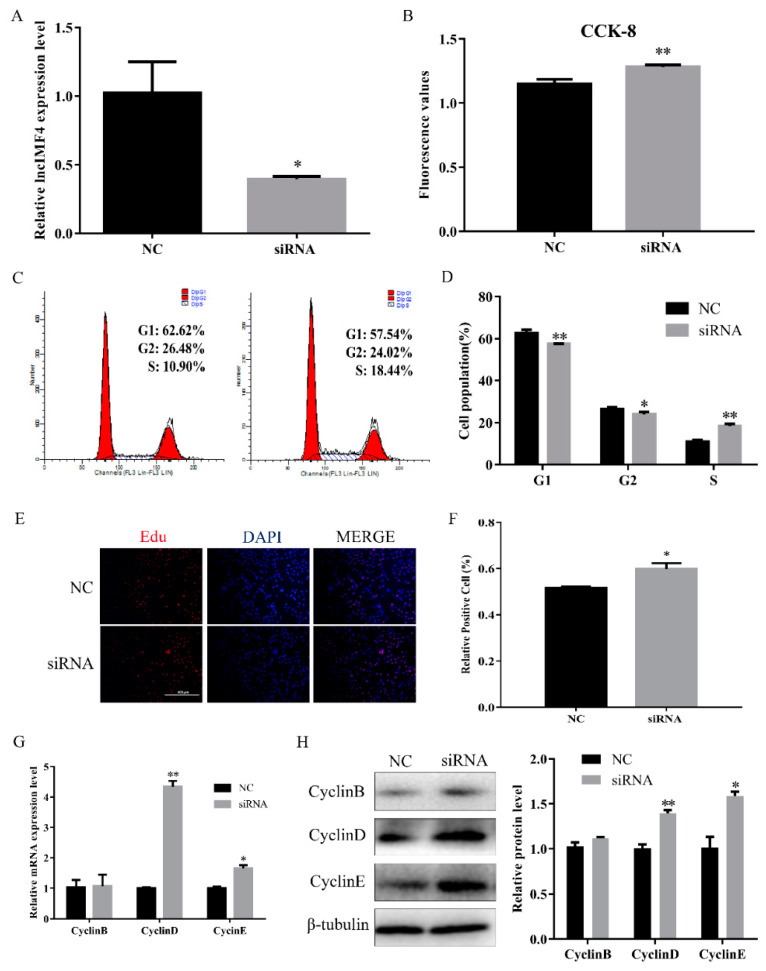
Knockdown of lncIMF4 promoted intramuscular adipocytes proliferation. LncIMF4 negative control (NC) or siRNA were transfected into cells at 40% density. (**A**) The inhibition efficiency of lncIMF4 in porcine intramuscular adipocytes treated with lncIMF4 siRNA. (**B**) Cell count was measured by Cell Counting Kit 8 (CCK8). (**C**) Cell cycle analyses. (**D**) The statistics results of cell cycle analysis. (**E**) EdU assay was performed after transfection for 24 h. (**F**) The percentage of EdU positive cells/DAPI positive cells was quantified. (**G**) RT-qPCR analyzed the cell cycle genes, cyclin B, cyclin D, and cyclin E after transfection for 24 h. (**H**) Western blot of the cell cycle genes, cyclin B, cyclin D, and cyclin E, after transfection for 24 h. The results were representative of means ± SEM of three independent experiments. *, *p* < 0.05; **, *p* < 0.01.

Proliferation is an important process for intramuscular adipose development. Therefore, we explored the function of lncIMF4 in the proliferation of intramuscular preadipocytes. We transfected siRNA into proliferating intramuscular preadipocytes to knockdown lncIMF4. The efficiency of siRNA meets the requirements of subsequent experiments (Figure 3A). CCK8 assay showed that the cell number was increased (Figure 3B). Furthermore, by cell cycle analysis, we found that knockdown of lncIMF4 increased the percentage of S phase cells and decreased the percentage of G1 and G2 phage cells (Figure 3C,D). The result of EdU assay was consistent with it (Figure 3E,F). Meanwhile, knockdown of lncIMF4 increased the levels of cell cycle-related genes compared with that of NC (Figure 3G,H). These results indicated that knockdown of lncIMF4 promoted the intramuscular adipocytes proliferation.

### 3.4. LncIMF4 Knockdown Promoted Intramuscular Adipogenic Differentiation

**Figure 4:**
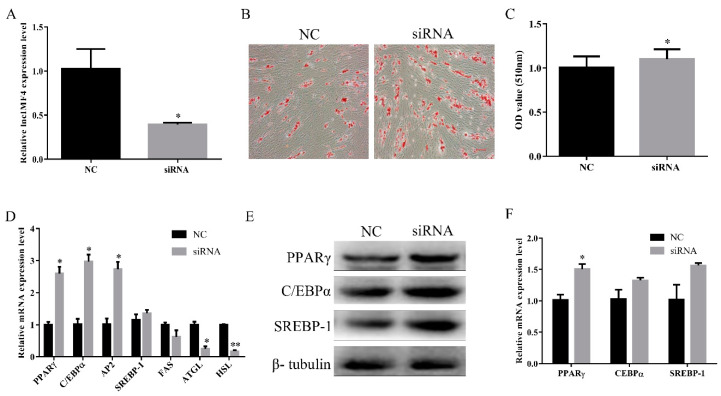
Knockdown of lncIMF4 promoted adipocytes lipogenesis. LncIMF4 siRNA or NC were transfected into cells at 70% density at 50 nM. (**A**) The inhibition efficiency of lncIMF4 in porcine intramuscular adipocytes after transfecting lncIMF4 siRNA with NC on 6 day of differentiation. (**B**) Oil Red O staining, scale bar = 100 µm. (**C**) Absorbance value at 510 nm after incubation with Oil Red O. (**D**) RT-qPCR analysis of adipogenic and lipolytic genes on 6 day of differentiation. (**E**) Western blot analysis of adipogenic genes on 6 day of differentiation. (**F**) The quantification of protein levels. The results were representative of means ± SEM of three independent experiments. *, *p* < 0.05; **, *p* < 0.01.

To explore the function of lncIMF4 during intramuscular adipocytes differentiation, we used the siRNA to knock down it, and the efficiency of siRNA reached the subsequence experiment requirement (Figure 4A). Oil Red O staining also showed a significant increase in intracellular lipid droplets in siRNA group (Figure 4B,C). Key genes of adipocytes differentiation were significantly increased after transfection with siRNA (Figure 4D). In addition, the protein levels of lipogenesis were upregulated (Figure 4E,F). Cumulatively, these results indicated that knockdown of lncIMF4 promoted adipocytes lipogenesis.

### 3.5. LncIMF4 Knockdown Inhibited Lipolysis by Attenuating Autophagy in Porcine Intramuscular Adipocytes

**Figure 5:**
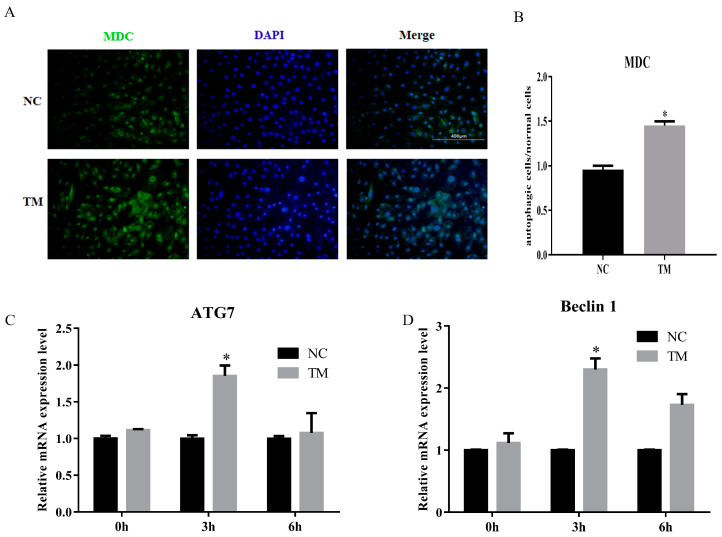
Autophagy model of porcine intramuscular adipocytes was constructed. (**A**) Monodansylcadaverine (MDC) staining of porcine intramuscular adipocytes after inducing autophagy by serum-free medium for 3 h. (**B**) The quantification of autophagic cells/normal cells. (**C**,**D**) RT-qPCR was carried out to detect the cell autophagic genes, ATG7 (**C**) and Beclin1 (**D**). Data were representative of means ± SEM of three independent experiments. *, *p* < 0.05. NC, negative control; TM, treatment.

In this study, we constructed the autophagy model of porcine intramuscular adipocytes by using nonserum culture medium after 6 days. The results showed that the number of autophagy cell was increased (Figure 5A,B). The levels of ATG7 and Baclin1 were also upregulated (Figure 5C,D). These results indicated that the autophagy model was successfully constructed.

**Figure 6:**
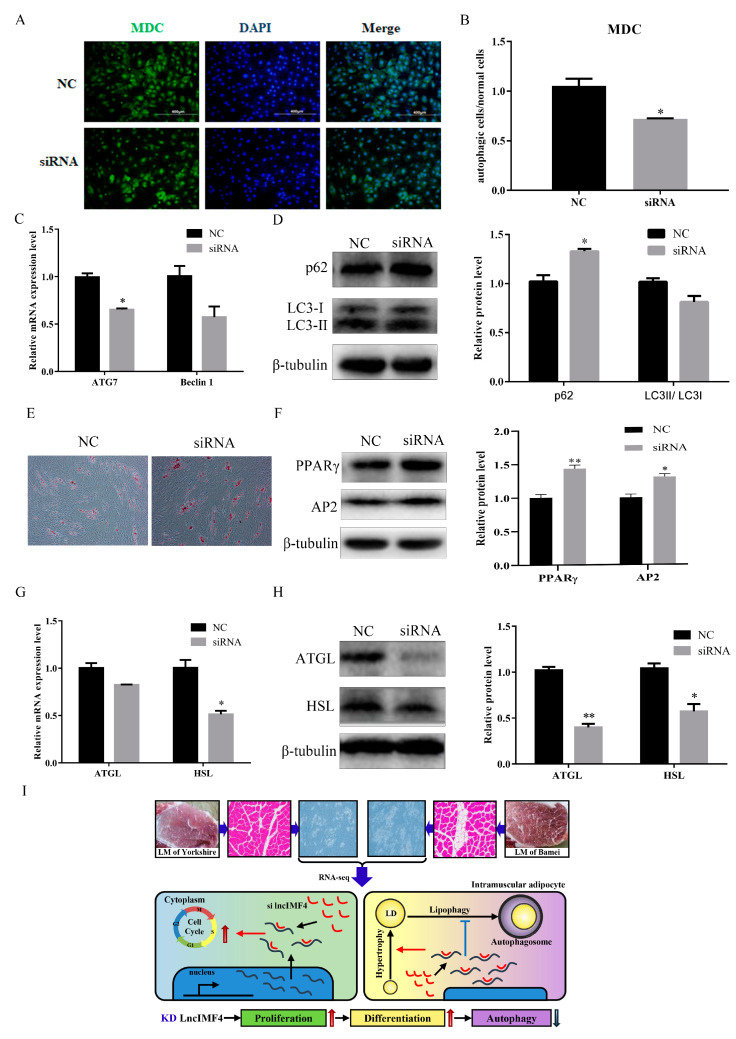
Knockdown of lncIMF4 inhibited lipolysis by downregulating autophagy. (**A**) MDC staining of porcine intramuscular adipocytes transfected into cells at 70% density at 50 nM after inducing autophagy by serum-free medium for 3 h. (**B**) The quantification of autophagic cells/normal cells. (**C**) RT-qPCR was carried out to detect the cell autophagic genes. (**D**) Western blot analysis of autophagic marker genes on 6 day of differentiation. (**E**) Oil Red O staining, scale bar = 100 µm. (**F**) Western blot analysis of adipogenic genes on 6 day of differentiation in autophagy model. (**G**) The mRNA expression level of lipolytic genes on 6 day of differentiation. (**H**) Western blot analysis and quantification of lipolytic protein levels. (**I**) The function of lncIMF4 in porcine intramuscular adipocytes. Data were representative of means ± SEM of three independent experiments. *, *p* < 0.05; **, *p* <0.01.

We predict that lncIMF4 is related to autophagy according to the results of GO term, which showed that lncIMF4 was enriched in autophagy-related pathway. Therefore, lncIMF4 were knockdown to test whether adipocytes autophagy was regulated. After transfection with siRNA, cells with green fluorescent dot particles were less than in the control group (Figure 6A,B). This indicated that autophagy was decreased when lncIMF4 was knocked down. The mRNA and protein levels of ATG7 and Baclin1 were consistent with it (Figure 6C). Meanwhile, the markers of autophagy, p62 were significantly upregulated and the LC3 were downregulated (Figure 6D). Furthermore, Oil Red O staining revealed that lncIMF4 knockdown promoted porcine intramuscular adipocyte differentiation in autophagy model (Figure 6E). Protein levels of PPARγ and AP2 showed the same result (Figure 6F). Consistently, the mRNA and protein levels of ATGL and HSL, the key genes of lipolysis, were both decreased after transfection with siRNA (Figure 6G,H). These results demonstrated that knockdown of lncIMF4 promoted porcine intramuscular adipocyte adipogenesis by attenuating autophagy. The function of lncIMF4 is shown in Figure 6I.

## 4. Discussion

Higher intramuscular fat content in pork plays an important role in improving meat quality [21]. Many factors are included in the biological processes. The regulatory role of lncRNA is an essential part of it [22]. In our previous study, we performed the lncRNA sequencing and found some lncRNAs related to adipogenesis. lncIMF4 was a novel lncRNA, which is predicted to participate in cell differentiation and autophagy. These processes could affect fat content in adipocytes [23,24,25]. This indicated that lncIMF4 have an effect on lipid synthesis in porcine intramuscular adipocyte. Interestingly, further research demonstrated that lncIMF4 was located both in nucleus and cytoplasm, so the role of it may be complex. In this study, we used small interference to explore its function in adipocytes.

The development of adipose tissue includes adipocytes proliferation and differentiation [26]. Recently, studies reflected that lncRNAs affect cell proliferation through variety of signaling [27,28]. In this study, we found that the level of lncIMF4 changed regularly along with cell proliferation. Based on this, we suspected that lncIMF4 participated in the regulation of proliferation of porcine intramuscular adipocyte. Subsequent experimental results were consistent with this hypothesis. Further study showed that knockdown lncIMF4 promoted intramuscular adipocyte proliferation. It reflected that lncIMF4 was important for intramuscular adipose development. The adipocyte differentiation is an important process regulating lipid deposition in adipose tissue. Adipocytes hyperplasia arises in the early stage of differentiation. Then, small lipid droplets appear in the cells and merge into large droplets after many adipogenic factors are activated. Finally, adipocytes become hypertrophic. LncRNA has been known as a regulator to affect adipocyte differentiation [29,30,31]. However, there is little research on the role of lncRNA in porcine adipocyte differentiation. In our study, GO term analysis showed that lncIMF4 is associated with cell differentiation. Furthermore, the level of lncIMF4 is related to fat deposition closely. Therefore, we explored the effects of lncIMF4 on porcine intramuscular adipocyte. Consistently, the results demonstrated that knockdown lncIMF4 promoted adipogenic differentiation in porcine intramuscular adipocyte. It confirmed that lncIMF4 regulated porcine intramuscular adipocyte lipogenesis.

Autophagy is a biological process that maintains life survival by degrading organelles and proteins when there is lack of nutrition, energy, or imbalance of environment. Defective autophagy, is associated with numerous diseases, including neurological disorders, cancer, cardiomyopathies and metabolic disorders [32]. Autophagy has a dilution effect on high intracellular lipids and influences lipid metabolism in many ways, from lipogenesis to lipolysis [33,34]. The autophagosomes will wrap lipids to ablate if autophagy occurs in adipocytes. Autophagy and lipolysis are both decreased with enough food supply, while both are increased in nutrient deprivation [35]. In this research, bioinformatics analysis predicts that lncIMF4 may have a particular regulatory effect on autophagy. Autophagy can affect the differentiation and adipogenic processes of adipocytes. Many signaling pathways were involved in these processes [36]. Therefore, we hypothesized that lncIMF4 regulated lipolysis to affect lipid deposition through autophagy. This is a new insight to study the relationship between lncRNA and intramuscular adipose. The autophagy of normal cells is very weak. To explore this regulation, we firstly constructed an autophagy model of porcine intramuscular adipocyte. In recent years, the induction of autophagy has focused on the construction of nutrient-deficient environment and drug treatment. To reduce stress on cells and reduce cell loss, autophagy was induced by starvation treatment in this study. The appropriate induction time was determined to be 3 h after the addition of serum-free medium, which was more transient than the time in which the adipocytes induced autophagy in other literature. Further study showed that in autophagy model, knockdown lncIMF4 promoted lipogenesis and inhibited lipolysis. Moreover, it also attenuated autophagy in porcine intramuscular adipocyte. Generally, we confirmed that lncIMF4 knockdown promoted lipogenesis of porcine intramuscular preadipocyte through attenuating autophagy. The underlying mechanism will be the focus of our next experiment.

## 5. Conclusions

In conclusion, we identified lncIMF4 as a novel lncRNA, which is related to intramuscular fat deposition. lncIMF4, located in both nucleus and cytoplasm, knockdown promoted proliferation and differentiation of porcine intramuscular adipocytes, whereas inhibited lipolysis through attenuating autophagy. This research provides a new perspective to study the role of lncRNA in regulating porcine intramuscular adipogenesis for promoting pork quality.

## Figures and Tables

**Table 1:** Primers used for real-time quantitative PCR.

Name	Forward (5′→3′)	Reverse (5′→3′)
PPARγ	AGGACTACCAAAGTGCCATCAAA	GAGGCTTTATCCCCACAGACAC
AP2	GAGCACCATAACCTTAGATGGA	AAATTCTGGTAGCCGTGACA
C/EBPα	CGATGCTCTTAGCTGAGTGT	GGTCCAAGAATTTCACCTCT
SREBP1	GGAGCCATGGATTGCACATT	GGCCCGGGAAGTCACTGT
Cyclin B	AATCCCTTCTTGTGGTTA	CTTAGATGTGGCATACTTG
MyoD	TACACCGACAACTCCATCCG	GAGGGCGGGTTGGAAATGAA
Cyclin E	CAGAGCAGCGAGCAGGAGC	GCAAGCTGCTTCCACACCACAT
FAS	CCCCGAATCTGCACTACCAC	AGTTGGGCTGAAGGATGACG
ATGL	TCACCAACACCAGCATCCA	GCACATCTCTCGAAGCACCA
HSL	CACTGACTGCTGACCCCAAG	TCCTCACTGTCCTGTCCTTCAC
ATG7	GATTGCCTGGTGGGTGGTAA	CATGGCTTTCGATGAGCTGC
Beclin1	AGTAGGTGAAGGCTAGGCGA	AGCTCGTGTCCAGTTTCAGG
GAPDH LncIMF4	AGGTCGGAGTGAACGGATTTG GTGGATTGGGAGCCTGCTAT	ACCATGTAGTGGAGGTCAATGAAG ACACTCCATGGCCTGGTAAAA
